# On the Insignificant Role of the Oxidation Process on Ultrafast High-Spatial-Frequency LIPSS Formation on Tungsten

**DOI:** 10.3390/nano11051069

**Published:** 2021-04-22

**Authors:** Priya Dominic, Florent Bourquard, Stéphanie Reynaud, Arnaud Weck, Jean-Philippe Colombier, Florence Garrelie

**Affiliations:** 1 UJM-Saint-Etienne, CNRS, Laboratoire Hubert Curien UMR 5516, Institute of Optics Graduate School, University Lyon, F-42023 St-Etienne, France; priya.dominic@univ-st-etienne.fr (P.D.); Florent.Bourquard@univ-st-etienne.fr (F.B.); stephanie.reynaud@univ-st-etienne.fr (S.R.); jean.philippe.colombier@univ-st-etienne.fr (J.-P.C.); 2 Department of Physics, University of Ottawa, STEM Complex, 150 Louis-Pasteur, Ottawa, ON K1N 6N5, Canada; aweck@uOttawa.ca; 3 Department of Mechanical Engineering, University of Ottawa, 161 Louis Pasteur, Ottawa, ON K1N 6N5, Canada; 4 Centre for Research in Photonics, University of Ottawa, 25 Templeton St, Ottawa, ON K1N 6N, Canada

**Keywords:** ultrafast laser nanostructuring, femtosecond laser, oxidation, sputtering, laser-induced periodic surface structures, high-spatial-frequency LIPSS, vacuum, Marangoni flow

## Abstract

The presence of surface oxides on the formation of laser-induced periodic surface structures (LIPSS) is regularly advocated to favor or even trigger the formation of high-spatial-frequency LIPSS (HSFL) during ultrafast laser-induced nano-structuring. This paper reports the effect of the laser texturing environment on the resulting surface oxides and its consequence for HSFLs formation. Nanoripples are produced on tungsten samples using a Ti:sapphire femtosecond laser under atmospheres with varying oxygen contents. Specifically, ambient, 10 mbar pressure of air, nitrogen and argon, and 10^−7^ mbar vacuum pressure are used. In addition, removal of any native oxide layer is achieved using plasma sputtering prior to laser irradiation. The resulting HSFLs have a sub-100 nm periodicity and sub 20 nm amplitude. The experiments reveal the negligible role of oxygen during the HSFL formation and clarifies the significant role of ambient pressure in the resulting HSFLs period.

## 1. Introduction

Ultrafast laser machining on material surfaces including metals [1,2], semiconductors [3,4,5], and polymers [6,7] to generate laser-induced periodic surface structures (LIPSS) has a broad spectrum of applications in biomedical surface engineering [8,9,10], tribology [11,12,13,14], color marking [15,16], memory devices fabrication [17,18,19], etc. Surface nanostructure formation can be mainly divided into (i) low-spatial-frequency LIPSS (**LSFLs**), with period λ/2 < Λ < λ (where λ is the laser wavelength) and (ii) high-spatial-frequency LIPSS (**HSFLs**), with period Λ < λ/2. Since the generation of LIPSS using a ruby laser by Birnbaum in 1965 [20], controversial theories have emerged over time to explain the formation of these nanostructures. One of the theories that explains the formation of LSFLs is the scattered wave model [21], where LSFLs are explained by the interference between a scattered surface wave or a polariton for metals (electromagnetic modes bound to and propagating along the surface) and the incoming laser wave. Sipe’s efficacy theory [22] analytically summarizes the generation of LSFLs on surface-roughness-induced inhomogeneous subsurface energy absorption and was confirmed by numerical simulations involving 3D finite-difference time-domain (3D FDTD) simulations [23]. These approaches can reproduce LSFLs formation based on an initial roughness layer, where topography centers induce a coherent superposition between the refracted wave and the far field scattered wave or the surface polaritons. Although these theories provide some insight into the formation of LSFLs, several theories have been proposed to explain all aspects associated with HSFLs, including second harmonic generation [24,25], changes in optical properties of the irradiated surface during the pulse [6], nascent plasma theory [26], surface instabilities leading to self-reorganization of matter upon laser irradiation [27], or hydrodynamics instabilities driven by electromagnetics [28].

One of the notable attempts in recent years to understand the formation of HSFLs is recognizing the associated role of oxidation. Öktem et al. [29] proposed a theory based on a feedback mechanism between oxide and nanostructure formation, where an initially positive feedback for nanostructure formation occurs by oxygen incorporation into the surface and is followed by a negative feedback as the thickness of this oxide layer increases, slowly breaking the nanostructure growth. Dostovalov et al. [30] introduced thermochemical LIPSS (TLIPSS), which are LIPSS formed due to metal oxidation rather than ablation. These TLIPSS have unique characteristics, including a rise in relief height, high degree of order, and an orientation parallel to the incident beam direction. The formation of TLIPSS on Ti, Ni, and Cr is explained based on a thermodynamic theory called Wagner theory [30,31]. The same group later recognized that HSFLs cannot be explained by Sipe’s theory and proposed an HSFLs formation mechanism based on different modes of propagation associated with scattered electromagnetic waves (SEWs), which evolve with a different percentage fraction of oxide [32].

Zuhlke et al. [33] and Peng et al. [34] demonstrated that structures like mounds formed on Ni and Ti surfaces after femtosecond laser irradiation have an oxide layer thickness ranging from nanometers to micrometers as confirmed by EDX and TEM characterizations. Later studies by Kirner et al. [35] showed that on Ti, hundreds of nanometers of oxide layer were found in association with both LSFLs and HSFLs, confirmed by µ-Raman, AES, and XPS analyses. Another investigation by Florian et al. [36] on a titanium alloy using glow discharge optical emission spectroscopy (GD-OES) for depth profiling the oxygen content in LSFLs showed a 200 nm thick oxide layer. One of the interesting conclusions of Florian et al. [37], based on FDTD simulations, is the requirement of 100 nm thick rough oxide layer for the formation of certain types of LSFL (with anomalous orientation) on CrN. However, the existence of sub 100 nm HSFLs with amplitude as small as a few tens of nanometers [26,38] may appear contradictory to previously mentioned theories.

Several HSFL formation theories point toward surface oxidation as a key parameter in understanding HSFL formation during ultrafast laser texturing. Oxygen can be incorporated from either one of the following sources, or both, during laser irradiation: (i) the native oxide layer, i.e., the adsorbed oxygen layer on the surface of materials with a thickness as low as 5 nm; (ii) the ambient air, which one might think is a major contributor.

In this work, the effect of laser processing environment on surface morphology was investigated to better understand HSFLs formation on tungsten. Various environments were used including high vacuum (10^−7^ mbar), argon (10 mbar), nitrogen (10 mbar), air (10 mbar), as well as ambient. Experiments were also carried out where the native oxide layer was removed by plasma sputtering, directly followed by laser irradiation. This study provides insight into the effect of oxygen on the formation of HSFLs. To date, only a very few attempts have been made to link processing environment to LSFLs or microspikes formation [39,40,41], conical microstructures [42], and HSFLs [43]. Here, HSFLs formation on tungsten (W) was investigated, because W is an important industrial metal (high hardness and high melting point) [44,45] and because it belongs to the same group as chromium in the periodic table, and thus W might be expected to undergo a similar oxidation-based HSFLs formation mechanism as discussed above. Furthermore, an investigation into the incorporation of oxygen during laser mater interaction is relevant not only to better understand HSFLs formation, but also because surface chemistry can influence many surface properties, including wettability [46,47,48], color [15,49,50], catalytic properties [51,52], and heat transfer potential [53,54], as seen for LSFLs.

## 2. Experimental Details

**Figure 1:**
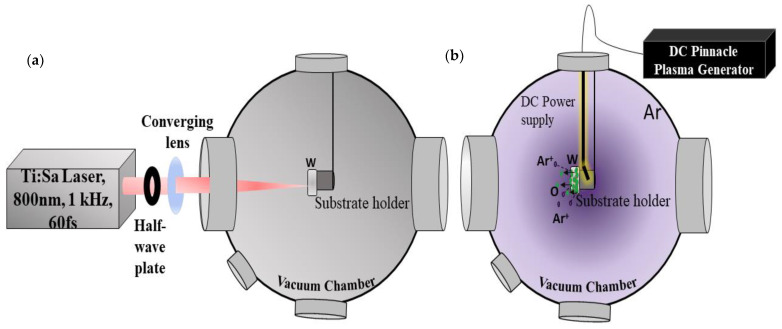
Experimental setup with the femtosecond laser beam focused into the vacuum chamber equipped with a plasma generator. (**a**) Irradiation of the W target inside a vacuum chamber. (**b**) Sputtering of the target surface prior to laser irradiation, in which the inert argon ion plasma created by polarizing the substrate holder using a continuous power supply provided by the pinnacle plus and the accelerated argon ions spray off the adsorbed oxide layer.

Polycrystalline tungsten samples (Goodfellow SARL, Lille, France) with dimensions 10 × 10 × 1 mm were electrochemically polished, resulting in an average roughness Ra of ~6 nm as measured with an AFM (Burker Dimension ICON, Billerica, MA, US). Polished tungsten samples were irradiated with a linearly polarized laser beam (Titanium-Sapphire, Legend Coherent Inc., Santa Jose, CA, USA) with a central wavelength of 800 nm, pulse duration of 60 fs, and 1 kHz pulse repetition rate, focused with a converging lens of focal length 30 cm to a focal spot size diameter of 100 µm into an ultrahigh vacuum chamber (Turbo pump, VINCI Technologies, Nanterre, France) equipped with a sputtering apparatus (DC pinnacle plus plasma generator, Denver, CO, USA), which can generate an argon ion plasma, as shown in Figure 1.

W surfaces were irradiated in different atmospheres including ambient, 10 mbar pressure of air, nitrogen (Air Products, Paris, France), argon (Air Products, Paris, France), and under high vacuum (10^−7^ mbar.). Tungsten peak ablation threshold fluence for a single pulse was 0.6 J/cm^2^, as calculated by Liu’s method [55]. The presented laser impacts were achieved with a peak fluence F_p_ = 0.35 J/cm^2^ and a number of pulses N = 25 in all environmental conditions, and the focal spot diameter of 100 μm was also evaluated near to these laser parameters by Liu’s method [55]. In order to eliminate the undesired native oxide layer, which could alter the photon-metal absorption process, argon ion sputtering was used (current = 0.25 A, frequency = 120 kHz, power = 16 W, voltage = 26 V, argon gas pressure = 10^−2^ mbar, and time of exposure to sputtering = 3 min). The process ensured the sputtering of any native oxide layer before irradiation with the laser and would thus reveal the role of the native oxide layer on HSFL formation. Sputtering was performed in the same vacuum chamber as the laser irradiation, thus ensuring no ambient air contamination of the sample surface. Surface morphology was characterized by SEM (FEI Nova NanoSEM 200, Hillsboro, OR, USA) and AFM.

## 3. Results and Discussion

**Figure 2:**
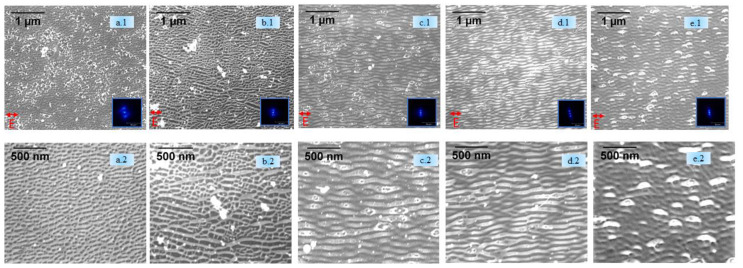

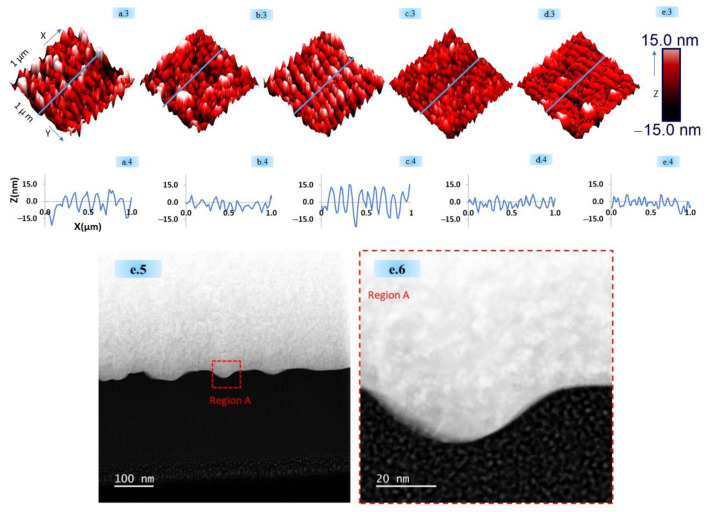
(**i**) **Top** (**a.1**–**e.2**) SEM images of HSFLs generated with no sputtering with FFT for (**a.1**,**a.2**) ambient, (**b.1**,**b.2**) air (10 mbar), (**c.1**,**c.2**) nitrogen (10 mbar), (**d.1**,**d.2**) argon (10 mbar), and (**e.1**,**e.2**) vacuum (10^−7^ mbar). (**ii**) **Middle** (**a.3**–**e.3**) AFM images (1 × 1 µm area) for (**a.3**) ambient, (**b.3**) air (10 mbar), (**c.3**) nitrogen (10 mbar), (**d.3**) argon (10 mbar), and (**e.3**) vacuum (10^−7^ mbar). (**iii**) (**a.4**–**e.4**) Line profiles corresponding to the AFM images indicated by the blue line (**a.4**,**b.4**,**c.4**,**d.4**,**e.4**). (**iv**) **Bottom** (**e.5**,**e.6**) STEM ADF cross-sectional images obtained for HSFLs formed in the vacuum. The ADF image shows several crests and valleys of HSFLs (**e.5**). A high-resolution image of Region A (**e.6**) confirms the amplitude of the HSFLs being sub 20 nm.

High-resolution SEM images of laser-irradiated samples without sputtering, taken at the center of the laser irradiated spots along with the corresponding FFT transforms (inset), are shown in Figure 2a.1,b.1,c.1,d.1,e.1,a.2,b.2,c.2,d.2,e.2. In the figure, HSFLs are observed to form parallel to the horizontally polarized femtosecond laser beam for all processing atmospheres. FFT analysis provided an HSFL period as small as 100 nm and AFM data, as presented in Figure 2a.4,b.4,c.4,d.4,e.4, confirmed this periodicity, and revealed the small amplitudes of the HSFL, varying from 10 to 20 nm. The scanning transmission electron microscope (STEM) annular dark field (ADF) cross-sectional images (Figure 2e.5,e.6) of the HSFLs formed in vacuum conditions agrees with the sub 20 nm amplitude obtained by AFM. The HSFL period (Λ) was 81 ± 2 nm, 92 ± 3nm, 109 ± 3 nm, 95 ± 3 nm, and 123 ± 2 nm for ambient, air (10 mbar), N (10 mbar), Ar (10 mbar), and vacuum (10^−7^ mbar), respectively.

**Figure 3:**
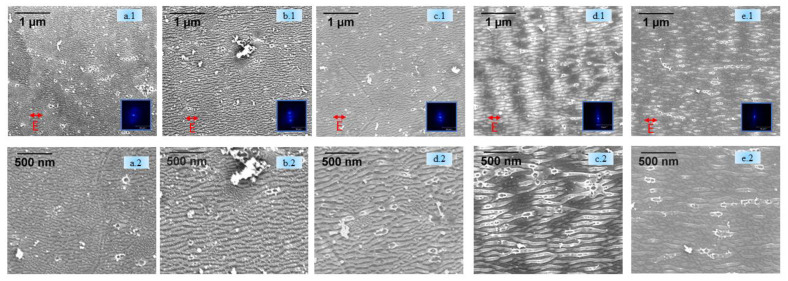

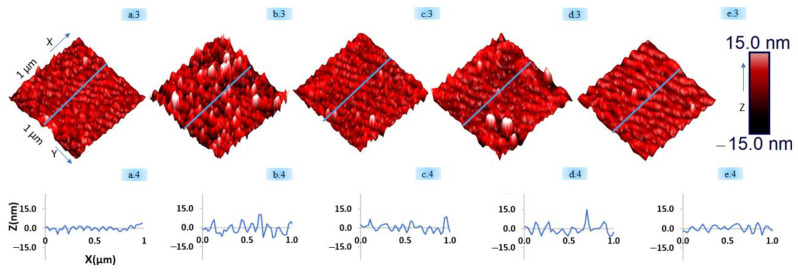
(**i**) **Top** (**a.1**–**e.2**) SEM images (row 1 and row 2, different magnifications) of HSFLs generated with sputtering with FFT for (**a.1**,**a.2**) ambient, (**b.1**,**b.2**) air (10 mbar), (**c.1**,**c.2**) nitrogen (10 mbar), (**d.1**,**d.2**) argon (10 mbar), and (**e.1**,**e.2**) vacuum (10^−7^ mbar). (**ii**) **Middle** (**a.3**–**e.3**) AFM images (1 × 1 µm area) for (**a.3**) ambient, (**b.3**) air (10 mbar), (**c.3**) nitrogen (10 mbar), (**d.3**) argon (10 mbar), and (**e.3**) vacuum (10^−7^ mbar). (**iii**) **Bottom** (**a.4**–**e.4**) Line profiles corresponding to the AFM images indicated by the blue line (**a.4**,**b.4**,**c.4**,**d.4**,**e.4**).

Figure 3a.1,b.1,c.1,d.1,e.1,a.2,b.2,c.2,d.2,e.2 shows the topography of the HSFLs formed after sputtering under the same various environments as those used in Figure 2. Here, the variation in periodicity was 51 ± 4 nm, 72 ± 5nm, 79 ± 3 nm, 88 ± 4 nm to 152 ± 5 nm, for ambient, 10 mbar air, 10 mbar N, 10 mbar Ar, and vacuum (10^−7^ mbar), respectively, which agrees with the periodicity obtained in the AFM profiles reported in Figure 3a.4,b.4,c.4,d.4,e.4. The amplitude of HSFL varied between 10 and 20 nm. 

**Figure 4:**
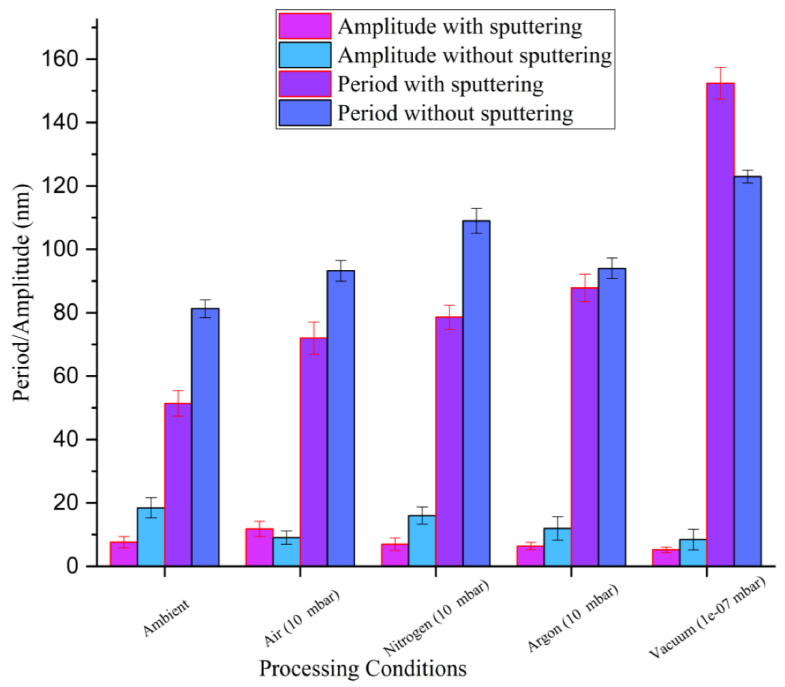
Variation in the period and amplitude of the HSFLs formed under different processing environments.

As evident from Figure 2 and Figure 3, the presence of HSFLs in high vacuum conditions and after sputtering confirms the insignificant role of atmospheric oxygen or any native oxide layer for the formation of these kind of laser-induced high-frequency structures. Upon analyzing Figure 4, we can observe that at a fixed pressure of 10 mbar, different processing atmospheres, whether air or in the presence of a non-reactive gas like argon or a reactive gas like nitrogen, yield HSFLs with almost the same features, thus again confirming the negligible contribution of the laser processing environment on the formation of HSFLs.

The variation in the HSFLs periods and amplitudes with different processing atmospheres, with and without sputtering, is shown in Figure 4. The general observed trend, with and without sputtering, is that the period of the HSFLs increases as the pressure decreases. As the ambient air was controlled to a pressure of 10 mbar (i.e., the condition of 10 mbar of air), the period increased compared with that of atmospheric pressure, and it further continued to increase as the pressure was reduced as low as 10^−7^ mbar (under vacuum). The decrease in ambient pressure can affect the hydrodynamical aspects of laser–matter interaction by influencing the surface tension of the molten liquid, thus leading to an increase in the period, as observed experimentally.

Rather than originating from SEWs in the laser-induced oxide layer, HSFLs are proposed to be established by molten material reorganization, driven by surface-tension-dependent transverse Marangoni gradient process [56]. The observed periodicity of the HSFLs (Λ) is related to the Marangoni instability as [28,55] $\Lambda =\frac{2пL}{\sqrt{Ma/8}}$, where *L* is the liquid layer thickness and *Ma* is a dimensionless quantity called the Marangoni number, as given by $Ma=-\frac{d\gamma }{dT\;}\frac{L\Delta T}{\mu D}$ [28,55], where dγ/dT is the temperature-dependent surface tension gradient, Δ*T* is the temperature difference, *µ* is the viscosity, and *D* is the thermal diffusivity. The dependence of the surface tension gradient on pressure (*P*) is given by $\frac{d\gamma }{dT}\propto -P$ [57]. From these equations, we obtain the HSFL period, Λ ∝ P ^−1/2^. This equation agrees with our experimental observations where smaller HSFL periods were observed at higher pressures, and supports a molten material reorganization process. The small HSFL period of approximately Λ–λ/8 can also be correlated to the confinement of the light absorption, as Marangoni instability provides Λ∝√L, where *L* is the liquid layer depth. Assuming that at this low fluence and high electron–phonon coupling strength, the electron thermal energy diffusion is weak, so the energy confinement can roughly be estimated by the optical penetration depth for tungsten at 800 nm. This is given by λ/4пk = 25 nm, where *k* = 2.9 is the extinction coefficient for photoexcited W [58]. For this small penetration depth, a small liquid layer, and thus small HSFL period, is expected.

The sputtering prior to laser irradiation tended to induce small changes in the periodicity for structures generated, as shown in Figure 4. This may be due to the change in roughness on the tungsten target induced by the sputtering process. It is known that the initial roughness can highly influence laser coupling and hence the resulting topography [59]. In particular, the concentration and kind of nanoreliefs as bumps and cavities resulting from the sputtering process affect the final period. It is expected that the introduction of additional scattering inhomogeneities on the surface with an average distance lower than the initial roughness would result in a higher concentration of scattering centers. This favors dipole–dipole coupling for nonradiative fields, reducing the pattern period [58]. Topographical features in the SEM (Figure 1 and Figure 2), for different gaseous atmospheres are different arising from the polycrystallinity of the samples. Because of the variation in energy absorption, phase transition, and lattice defects storage for different crystal orientations, their interaction with the laser beam can be different [60]. Because of the lower fluence used for the generation of HSFLs, these changes can be more intense.

All observations in this work signal that the formation of HSFLs for a given fluence and number of pulses under different laser processing atmospheres is mostly independent of any supply of oxygen. These results strongly suggest that HSFLs are more likely to be explained by theories based on electromagnetics coupling with hydrodynamical concepts of laser matter rather than the propagation modes implying oxidation models. More specifically, inhomogeneous subsurface energy absorption arising from the local non-radiative electromagnetic response can trigger nanoconvective instabilities. These thermoconvective instabilities mediated by temperature-gradient-dependent flow of capillary waves and their further solidification producing nanostructures [28,55,61] can explain the HSFL formation observed in the absence of oxygen and the decrease in HSFLs period with the ambient pressure.

## 4. Conclusions

Femtosecond-laser-induced HSFLs with sub 100 nm period and sub 20 nm amplitude were achieved on tungsten with a given fluence (Fp = 0.35 J/cm^2^) and number of laser pulses (N = 25) under different processing atmospheres, namely ambient, air (10 mbar), nitrogen (10 mbar), argon (10 mbar), and vacuum (10^−7^ mbar) with and without sputtering. This points toward neither ambient oxygen nor the native oxide layer playing a significant role in the formation of HSFLs. The experiment under different atmospheres showed how HSFLs can be obtained without any external supply of oxygen. They can be achieved in a chamber almost devoid of any gases with a pressure as low as 10^−7^ mbar, or in the presence of a nonreactive gaseous atmosphere like argon, or even on the presence of a reactive gas like nitrogen. Laser processing experiments immediately after plasma sputtering of the tungsten surface yielded HSFLs with no native oxide layer. The generation of HSFLs is mostly dependent upon material properties like surface roughness, grain orientation, and laser parameters; hence, oxygen as a necessary condition can be neglected. SEM, FFT, and AFM data provided complementary information regarding the topography of HSFLs. The decrease in HSFL periodicity with increasing pressure may be understood in terms of the influence of pressure on the Marangoni flow of the molten liquid. This supports a hydrodynamic origin of HSFLs formation where the observed period can be tuned by the pressure dependence of the thermo-capillarity process. This work paves the way toward the control of 100 nm features by changing the pressure and chemical environment.

## Data Availability

Not applicable.
